# *Not*I Microarrays: Novel Epigenetic Markers for Early Detection and Prognosis of High Grade Serous Ovarian Cancer

**DOI:** 10.3390/ijms131013352

**Published:** 2012-10-18

**Authors:** Vladimir Kashuba, Alexey A. Dmitriev, George S. Krasnov, Tatiana Pavlova, Ilya Ignatjev, Vasily V. Gordiyuk, Anna V. Gerashchenko, Eleonora A. Braga, Surya P. Yenamandra, Michael Lerman, Vera N. Senchenko, Eugene Zabarovsky

**Affiliations:** 1 Department of Microbiology, Tumor and Cell Biology, Karolinska Institute, Stockholm SE-171 77, Sweden; E-Mails: Vladimir.Kashuba@ki.se (V.K.); tatiana.pavlova@ki.se (T.P.); ivigi@mail.ru (I.I.); suryay@bii.a-star.edu.sg (S.P.Y.); 2 Department of Molecular Oncogenetics, Institute of Molecular Biology and Genetics, NASU, Kiev 01000, Ukraine; E-Mails: vasilij_gordiyuk@yahoo.com (V.V.G.); geranru@yahoo.com (A.V.G.); 3 Laboratory of Structural and Functional Genomics, Engelhard Institute of Molecular Biology, Russian Academy of Science, Moscow 119991, Russia; E-Mails: Alex_245@mail.ru (A.A.D.); gskrasnov@mail.ru (G.S.K.); versen@eimb.ru (V.N.S.); 4 Russian State Genetics Center GosNIIgenetika, Moscow 117545, Russia; E-Mail: ebraga@genetika.ru; 5 Affina Biotechnologies, Stamford, CT 06902, USA; E-Mail: lermanmi@gmail.com; 6 Linköping University, Department of Clinical and Experimental Medicine, Linköping SE-581 85, Sweden

**Keywords:** ovarian cancer, biomarkers, *Not*I microarrays, epigenetics, early detection of ovarian cancer, prognosis of ovarian cancer

## Abstract

Chromosome 3-specific *Not*I microarray (NMA) containing 180 clones with 188 genes was used in the study to analyze 18 high grade serous ovarian cancer (HGSOC) samples and 7 benign ovarian tumors. We aimed to find novel methylation-dependent biomarkers for early detection and prognosis of HGSOC. Thirty five *Not*I markers showed frequency of methylation/deletion more or equal to 17%. To check the results of NMA hybridizations several samples for four genes (*LRRC3B*, *THRB*, *ITGA9* and *RBSP3* (*CTDSPL*)) were bisulfite sequenced and confirmed the results of NMA hybridization. A set of eight biomarkers: *NKIRAS1/RPL15*, *THRB*, *RBPS3 (CTDSPL)*, *IQSEC1*, *NBEAL2*, *ZIC4*, *LOC285205* and *FOXP1*, was identified as the most prominent set capable to detect both early and late stages of ovarian cancer. Sensitivity of this set is equal to (72 ± 11)% and specificity (94 ± 5)%. Early stages represented the most complicated cases for detection. To distinguish between Stages I + II and Stages III + IV of ovarian cancer the most perspective set of biomarkers would include *LOC285205*, *CGGBP1*, *EPHB1* and *NKIRAS1/RPL15*. The sensitivity of the set is equal to (80 ± 13)% and the specificity is (88 ± 12)%. Using this technique we plan to validate this panel with new epithelial ovarian cancer samples and add markers from other chromosomes.

## 1. Introduction

Epithelial ovarian cancer (EOC) remains a highly lethal malignancy. It is the fifth leading cause of cancer deaths among women in the United States (22,000 new cases and 16,000 deaths annually) and causes more than 140,000 deaths annually in women worldwide. Despite intensive research efforts over the past decade directed toward improved detection and treatment of ovarian cancer, the majority of women diagnosed with ovarian cancer die from the disease [1].

The epithelium is the tissue where most ovarian cancers arise [2]. Ovarian cancer is classified into several stages according to the American Joint Committee on Cancer/Tumor Node Metastasis (AJCC/TNM) and International Federation of Gynecology and Obstetrics (FIGO) staging systems which are based on how far the cancer has spread. In Stages I and II, the tumor is confined to the ovaries, while there is local metastasis (usually lymph) in Stage III and there is distal organ metastases in Stage IV [3].

Currently, the two principle obstacles in treating this life threatening disease are lack of effective biomarkers for early detection and drug resistance after initial chemotherapy.

Due to the atypical syndrome of the early stage of ovarian cancer, it is difficult to diagnose in its early stages. EOC (90% of ovarian cancer) is diagnosed at an advanced Stages III and IV in 75% of all cases, where the disease has spread throughout the abdomen. Patients with advanced stage disease have a 5-year survival of only 30% in contrast to early-stage disease (confined to the ovaries), where 5-year survival exceeds 80% [4].

Ovarian cancer is a heterogeneous disease both histologically and in patterns of disease progression. EOC is comprised of four major histologic subtypes: endometrioid, mucinous, clear cell and serous ovarian cancer. The high grade variant of serous ovarian cancer (HGSOC) is typically diagnosed in late stage, and is mostly responsible for the high lethality rate of ovarian cancer. It is also the subtype with the highest prevalence, estimated at ~70% of all cases [5,6].

Similar to other cancers, the initiation and development of ovarian cancer is characterized by activation of oncogenes and disruption of tumor suppressor genes (TSGs) by both genetic and epigenetic mechanisms. While it is well known that it is challenging to treat ovarian cancer through a genetic strategy due in part to its heterogeneity, the reversibility of epigenetic mechanisms involved in ovarian cancer opens exciting new avenues for treatment. The epigenomics of ovarian cancer has therefore become a rapidly expanding field leading to intense investigation.

Hypermethylation of CpG islands in gene promoter regions has been observed as a frequent mechanism associated with inactivation of TSGs which contributes to malignant transformation. As aberrant methylation is thought to be one of the earliest observable molecular changes in carcinogenesis, the detection of alterations in DNA methylation patterns has potential applicability to the detection of early-stage or pre-malignant disease [7,8]. Specific methylated DNA markers can be detected in the serum, plasma and peritoneal fluid of ovarian cancer patients [9].

Thus, cancer epigenetic studies hold great promise in revealing potent biomarkers for improved cancer detection [10,11]. Candidate gene and whole-genome studies have identified methylation signatures that may serve as biomarkers for EOC characterization including classification [7], progression [12] and response to therapy [13].

We constructed a new generation (~20 years ago) of lambda based cloning vectors [14]. These vectors opened new possibilities for gene cloning and analysis. Using these vectors we invented new approaches for construction of *Not*I linking and jumping libraries that have several advantages compared to previous techniques; they enabled efficient construction of such libraries representative and in plasmid form [15,16]. We experimentally confirmed that there is a direct association between CpG islands, *Not*I sites and expressed sequences in the human genome [17,18]. We constructed numerous linking libraries with different restriction enzymes in an attempt to generate representative *Not*I linking libraries, covering the whole human genome [19]. We generated more than 100,000 *Not*I flanking sequences and identified among them approximately 22,000 unique *Not*I sequences comprising 17 Mb information [20,21]. It was believed at this time that human genome contains only 3100 *Not*I sites and we showed for the first time that there are more than 10,000 NotI sites.

**Figure 1:**
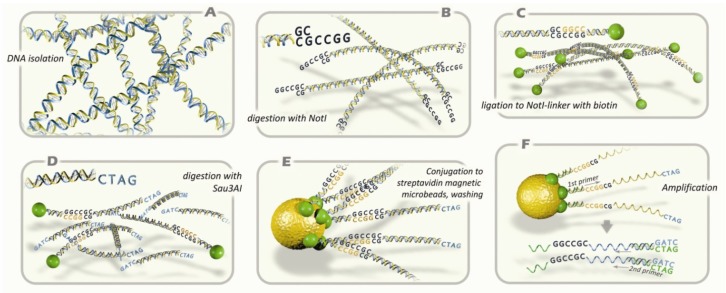
Principal scheme of *Not*I microarray analysis protocol. (**A**) Isolation of genomic DNA; (**B**) digestion with methyl-specific rare-cutter enzyme *Not*I; (**C**) ligation of fragments with *Not*I-linker containing biotin; (**D**) digestion with 4-base pair recognizing restriction enzyme Sau3AI; (**E**) conjugation to microbeads containing streptavidin; washing; (**F**) amplification of DNA sequences that has been attached to microbeads. The standard procedures are performed: microarray hybridization, cloning, and sequencing analysis.

With this sequencing information we suggested to construct and to use *Not*I microarrays (NMA, see Figure 1) for comparison of normal and malignant cells [22]. Here we present our results of analysis using NMA of 18 HGSOC and 7 benign ovarian adenomas (BOA) and developed a set of novel epigenetic biomarkers for early detection and diagnosis of EOC.

## 2. Results

### 2.1. Analysis of Methylation Frequency Using *Not*I Microarrays

**Figure 2:**
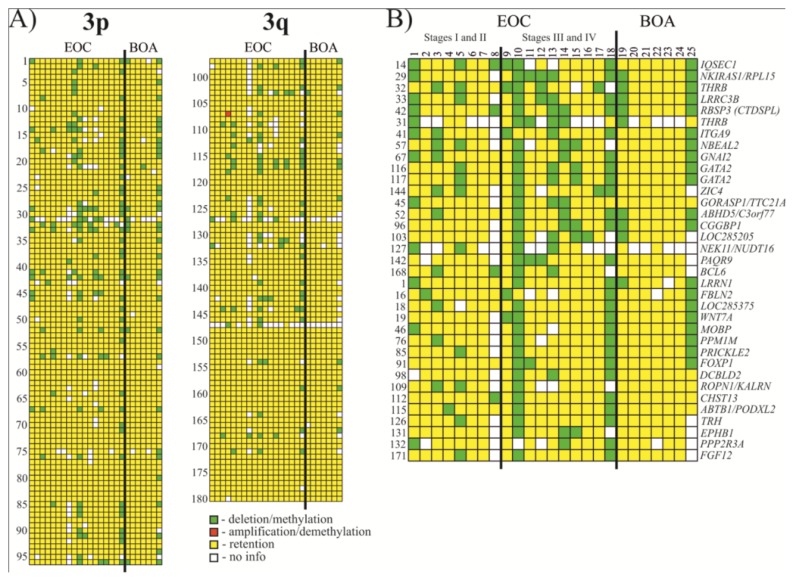
Hybridization pattern of DNA from Epithelial ovarian cancer (EOC) and benign ovarian adenomas (BOA) samples on *Not*I-microarrays. (**A**) Vertically, 180 *Not*I sites arranged according to their localization on chromosome 3 (from 3p26.2 to 3p11.1 and from 3q11.2 to 3q29). Horizontally, 25 ovarian samples (18 EOC and 7 BOA); (**B**) Vertically, 35 *Not*I sites arranged by methylation/deletion frequency (from 33% to 17%).

Thirty five *Not*I markers showed frequency of methylation/deletion more or equal to 17% (see Figure 2 and Tables 1 and S1). Among the most affected genes were *IQSEC1*, *NKIRAS1/RPL15*, *THRB*, *LRRC3B* and *RBSP3 (CTDSPL)* that showed 33% methylation/deletion (up to 38% when not counting samples with “no information”).

**Figure 3:**
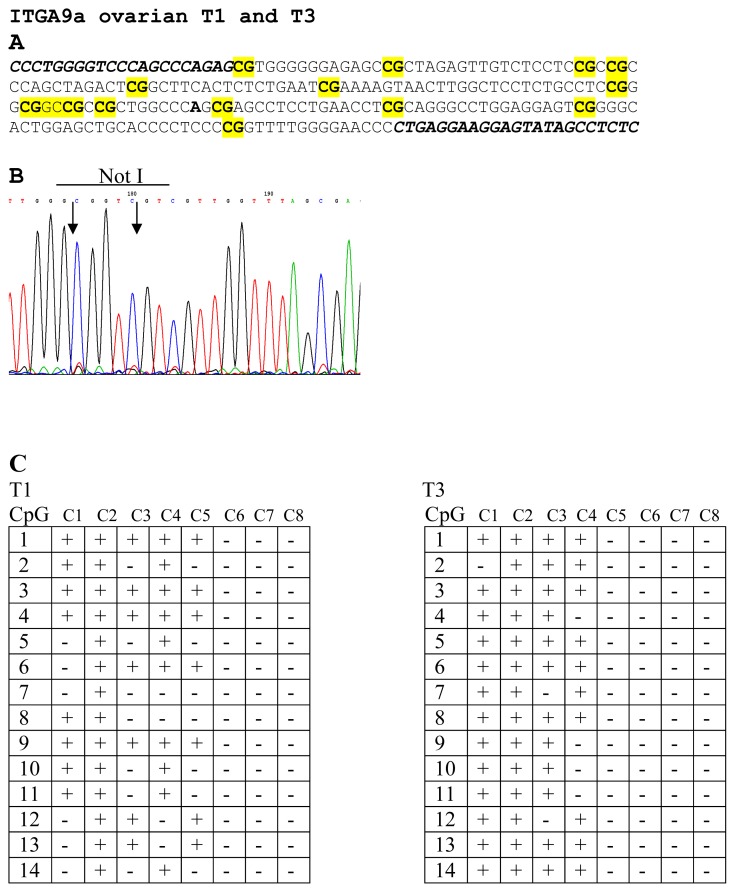
Bisulfite sequencing of *ITGA9* in EOC samples. CG-pairs containing methylated cytosine are shown in bold and yellow (**A**). Primers for bisulfite sequencing (A) are shown in italics below example of sequencing diagrams (**B**) demonstrating methylated sequence of *ITGA9* is shown. In the two tables (**C**) methylated (+) and unmethylated (−) CG pairs are shown in eight sequenced clones for T1 and T3 samples.

To prove the results of NMA hybridizations, several samples were sequenced. For genes *LRRC3B* (No. 12 and 13) and *THRB* (No. 9 and 10) were selected two samples and both of them were found methylated. For *ITGA9* three samples were selected (No. 1, 2 and 3) and only sample No. 2 was found unmethylated. For *RBSP3* (*CTDSPL*) three samples were also selected (No. 14, 15 and 16) and as it follows from the Figure 1B only one sample was found to be methylated (No. 14). Thus results of bisulfite sequencing confirmed the results of NMA (see Figure 3).

Eight genes showed the tendency to increase methylation/deletion frequency during ovarian cancer progression (stages III + IV relative to stages I + II, see Table 2).

### 2.2. Selection of Genes/Biomarkers for Detection and Discrimination EOC with Different Histological Characteristics

For detection cancer in ovarian biopsy on all stages including early one of the most perspective set from analyzed genes included 8 biomarkers: *NKIRAS1/RPL15*, *THRB*, *RBPS3* (*CTDSPL*), *IQSEC1*, *NBEAL2*, *ZIC4*, *LOC285205* and *FOXP1*. If methylation/deletion was found in two or more of these biomarkers then sample would be recognized as cancer. Sensitivity of this set is equal (72 ± 11)% and specificity (94 ± 5)%. Early stages represented the most complicated cases for detection.

BOA samples had no changes in five cases from seven analyzed, so in order to distinguish them from cancer samples it is possible to use the same set as for cancer detection. If methylation/deletion was found in two or more of the above-mentioned biomarkers then the sample would be recognized as cancer. Sensitivity of this set is equal (72 ± 11)% and specificity (71 ± 17)%.

To distinguish between Stages I + II and Stages III + IV of ovarian cancer the most perspective set would include *LOC285205*, *CGGBP1*, *EPHB1*, and *NKIRAS1/RPL15* biomarkers. If we found methylation/deletion in 1 or more of these biomarkers then sample would be recognized as a sample from III + IV stages. In this case the sensitivity of the set is equal to (80 ± 13)% and the specificity is (88 ± 12)%. Stages III + IV methylation/deletion assumed as positive result and Stages I + II as negative.

In summary, the suggested set of 10 markers (*NKIRAS1/RPL15*, *THRB*, *RBPS3* (*CTDSPL*), *IQSEC1*, *NBEAL2*, *ZIC4*, *LOC285205*, *FOXP1*, *CGGBP1*, *EPHB1*) would allow us to discriminate/diagnose the majority of EOC cases with sensitivity and specificity higher than 71% (up to 94%) (Tables 3 and 4).

## 3. Discussion

In this work we used chromosome 3 specific *Not*I-microarrays with 180 clones containing 188 genes to analyze 18 HGSOC and 7 BOA samples. The main idea of the approach is that NotI enzyme cuts only unmethylated CpG pairs inside the recognition site of the enzyme (5′-G**CG**GC**CG**C-3′) and only small fraction (0.1%–0.05%) of the human genome containing *Not*I digested fragments is labeled. The NR probes were prepared as described earlier (see Materials and Methods and [84,103–105]). Thus, in contrast to all other methods where undigested by methylation sensitive enzymes DNA fragments are labeled, we label only digested DNA fragments. As a consequence, our probe contains 10-fold less repeats, it is hotter, not very sensitive to incomplete digestion and gives less background. To confirm results of NMA hybridizations, several samples for four genes, namely, *LRRC3B*, *THRB*, *ITGA9* and *RBSP3* (*CTDSPL*) were sequenced (see Figure 3). All the genes were found to be methylated. It is important to note that for bisulfite sequencing we always clone PCR product and then sequence 8 clones. Of course, we could not exclude the possibility that unmethylated clones, *i.e.*, green in Figure 2, were in fact deleted. However, this issue can be considered as an advantage when search for TSGs is being performed: TSGs are inactivated either by methylation or deletions, or both of them, and we can detect these events simultaneously. If it is necessary, discrimination between these changes can be done using e.g., qPCR, bisulfite sequencing, and using another NR probe prepared with tumor DNA after amplification with Phi29 DNA polymerase.

This study clearly demonstrated that *Not*I-microarrays are powerful tools to find methylated genes and resulted in identification of many novel genes/biomarkers that can be important for the development of more specific biomarker sets for early diagnosis and new approaches to therapy of EOC.

As it was mentioned in the Introduction the major problems in HGSOC are its early diagnosis, discrimination between Stages I + II and Stages III + IV and absence of specific molecular markers.

For example, ovarian cancer screening with transvaginal ultrasound (TVU) and CA125 was evaluated in the Prostate, Lung, Colorectal, and Ovarian (PLCO) trials. However, it was revealed that the predictive value of both tests was relatively low [106]. Of 39,115 women randomized to receive screening, 28,816 received at least 1 test. Abnormal TVU was found in 1338 (4.7%), and abnormal CA-125 in 402 (1.4%). Twenty-nine neoplasms were identified (26 ovarian, 2 fallopian, and 1 primary peritoneal neoplasm). Nine were tumors of low malignant potential and 20 were invasive. The positive predictive value for invasive cancer was 3.7% for an abnormal CA-125, 1.0% for an abnormal TVU, and 23.5% if both tests were abnormal. The authors concluded that nothing in the findings reported in the paper suggests a need to revise the present (1996) ovarian cancer screening guidelines of the US Preventive Services Task Force, which state “routine screening for ovarian cancer by ultrasound, the measurement of serum tumor markers, or pelvic examination is not recommended.”

Increasing evidences indicate that epigenetic mechanisms may play a major role in the development of ovarian cancer [2]. Aberrant DNA methylation occurs commonly in tumors and is considered to be one of the earliest molecular changes in carcinogenesis [8,107–110]. Furthermore, studies have identified tumor-specific gene methylation in blood DNA of patients with EOC [9,111–113], indicating that methylation patterns in plasma DNA have potential to serve as non-invasive biomarkers of EOC.

Thus, Caceres *et al.* [9] using the panel of 6 TSGs: *BRCA1*, *RASSF1A*, *APC*, *p14^ARF^*, *p16^INK4A^* and *DAP-Kinase*, demonstrated that promoter hypermethylation is common in ovarian cancer, including stage I disease, and can be readily detected in a specific manner in serum and peritoneal fluid DNA. In this initial feasibility study, they observed a sensitivity of 82% and 100% specificity in serum. However, hypermethylation was observed in all histologic cell types, grades, and stages of ovarian tumor examined and moreover TSGs that were used in the study were not ovarian cancer specific.

In the study by Melnikov *et al.* [114] previously developed microarray-based technique was used; authors evaluated differences in DNA methylation profiles in a panel of 56 genes using sections of serous papillary adenocarcinomas and uninvolved ovaries (*n* = 30) from women in a high-risk group. Methylation profiles were also generated for circulating DNA from blood of patients (*n* = 33) and healthy controls (*n* = 33). Using the most differentially methylated genes for naïve Bayesian analysis, they identified 10 of these profiles as potentially informative in tissues. Various combinations of these genes produced 69% sensitivity and 70% specificity for cancer detection as estimated under a stratified, fivefold cross-validation protocol. In plasma, five genes were identified as informative; their combination had 85% sensitivity and 61% specificity for cancer detection. These results suggest that differential methylation profiling in heterogeneous samples has the potential to identify components of a composite biomarker that may detect ovarian cancer in blood with significant accuracy.

Ten of the most well-known methylated genes in ovarian cancer were selected in Montavon *et al.*, 2012 study: *BRCA1*, *CDH1*, *DLEC1*, *EN1*, *GATA4*, *GATA5*, *HOXa9*, *HSULF1; RASSF1A* and *SFN*. Although some of them showed very high frequency of methylation in HGSOC (e.g., *SFN* 100%) and *HOXa9* and *EN1* showed sensitivity of 98.8% and specificity of 91.7%, none of the markers after correction for multiple testing, gene methylation was not significantly associated with any clinico pathological characteristics including discrimination of Stages I + II from Stages III + IV [6].

It has become clear that even with the great promise of DNA methylation biomarkers in epithelial ovarian cancer, the identification of highly specific, sensitive and robust panels of markers and the standardization of analysis techniques are still required in order to improve detection, treatment and thus patients’ outcome [115].

Among 11 genes included in our set there are six genes for which no information about their involvement in ovarian carcinogenesis have been shown: *NKIRAS1/RPL15*, *THRB*, *IQSEC1*, *NBEAL2* and *ZIC4* (see Table 4). However, for all 11 genes in the set it was shown involvement in some of cancers. In some cases it was shown only decreased expression (*LOC285205*, *EPHB1* and *RBSP3* (*CTDSPL*)) and in other cases loss of heterozygosity and copy number changes (*CGGBP1* and *RBSP3* (*CTDSPL*)) in ovarian cancer. Some other genes showed decreased expression in other cancer types, like *EPHB1* in gastric carcinoma, *NKIRAS1* in kidney cancer, *THRB* in many cancers, etc. *FOXP1* is found to be significantly down-regulated in stage III serous ovarian carcinoma (see Table 4). Among two genes from the Table 2 not included in the set is the well-known tumor suppressor *WNT7A*. WNT (Wingless-Type Mouse mammary tumor virus Integration Site Family) growth factors have diverse roles in governing cell fate, proliferation, migration, polarity, and death in multicellular organisms. *WNT7A* has been demonstrated to be a TSG in lung cancer. Normally WNT7A maintains epithelial differentiation and inhibits growth of the transformed cell in a subset of human Non-Small Cell Lung Cancer (NSCLC). It was shown that WNT7A regulates tumor growth and progression in ovarian cancer. *GATA2* is also involved in the development of bladder and breast cancer and acute myelogenous leukemia. Other genes were also shown to be involved in the process of carcinogenesis (see Table 4). Among the genes represented in the set there are genes encoding transcriptional regulators (*CGGBP1*, *GATA2*), receptors (*EPHB1*, *THRB*), phosphatase (*RBSP3* (*CTDSPL*)), proteins interacting with other proteins (*NKIRAS1*, *IQSEC1*, *NBEAL2*, *ZIC4*).

These proteins are involved in different cancer-related pathways:

EPHB1 for example participates in the Ephrin-EphR Signaling Pathway. In this pathway members of RAS family, MAPK, oncogene NCK1 and other genes important for cell developmental processes are included. Ephrin receptors make up the largest subgroup of the receptor tyrosine kinase (RTK) family. The protein encoded by this gene is a receptor for ephrin-B family members.
NKIRAS1 is a potent regulator of NF-κ-B activity by preventing the degradation of NF-κ-B inhibitor beta (NFKBIB) by most signals, explaining why NFKBIB is more resistant to degradation.
IQSEC1 links epidermal growth factor receptor signaling to ARF6 activation to induce breast cancer invasion (see Table 4).
RBSP3 (CTDSPL) is a potential activator of the RB1 gene pathway [81].

At the same time little is known about function and involvement of *LOC285205* and *CGGBP1* genes in carcinogenesis. Thus this novel set of markers open perspectives for further improvement in early detection and diagnosis of EOC. Of course, this set needs further validation and testing with new samples of ovarian cancer including blood samples. Also this panel should be enriched with additional markers from other chromosomes; we are planning to construct NMA containing 10,000–15,000 genes.

## 4. Materials and Methods

### 4.1. Tissue Specimens

Eighteen paired specimens of epithelial ovarian carcinoma (all the samples were HGSOC) and seven cases of benign ovarian adenoma (BOA) were obtained after surgical resection of primary EOC or adenoma prior radiation or chemotherapy and stored in liquid nitrogen. “Normal” controls were obtained minimum at 2 cm distance from the tumor and confirmed histologically as normal ovarian epithelial cells. The diagnosis was verified by histopathology and only samples containing 70%–80% or more tumor cells were used in the study. The samples were collected in accordance to the guidelines issued by the Ethics Committee of Blokhin Cancer Research Center, Russian Academy of Medical Sciences (Moscow). All patients gave written informed consent that is available upon request. The Ethics Committee of Blokhin Cancer Research Center, Russian Academy of Medical Sciences specifically approved this study. The study was done in accordance with the principles outlined in the Declaration of Helsinki. All tumor specimens were characterized according to the International System of Clinico-Morphological Classification of Tumors, based on the tumor-node-metastasis (TNM) and staging classification of 2002 and AJCC/TNM criteria classification of 1999 [3].

### 4.2. NotI-Microarrays

One hundred and eighty *Not*I linking clones from human chromosome 3 containing 188 genes [22,116] with inserts up to 15 kb were immobilized on the glass slides in six replications. Plasmid DNA for immobilization on the glasses was isolated with a HiPure Plasmid Midiprep kit (Invitrogen) and printed on the silanized glasses at a concentration of 0.25 μg/μL with a QarrayMini microarrayer (Genetix, UK). DNA from *E. coli* was used as negative hybridization control.

### 4.3. NotI Probes and Hybridization

Preparation of *Not*I representations (*Not*I probes, NR, see Figure 1) with special labeling procedure, where only sequences surrounding *Not*I sites were labeled (0.1%–0.05% of the total human genomic DNA) was done using paired normal (adjacent control) and tumor DNA essentially as described previously [84,103–105]. In brief, this involved DNA digestion with *Not*I restriction enzyme, ligation to *Not*I-linkers with biotin, digestion with Sau3A restriction enzyme, immobilization on Dynabeads M-280 Streptavidin “Dynal” and finally washing and ligation to DNA bound with magnetic beads with Sau3A-linkers. The enriched DNA was amplified by PCR using universal and linker-primers. PCR conditions were the following: 2 min at 95 °C, then 35 cycles of denaturation (45 s at 95 °C), annealing (40 s at 64 °C) and synthesis (2 min 20 s at 72 °C). Thereafter, 200–400 ng of NR was labelled by PCR as described above but in the presence of 1.25 nM of Cy5-dCTP (or Cy3-dCTP). This probe detected both deleted/amplified and methylated/unmethylated sequences. If it is necessary, discrimination between these changes can be done using qPCR, bisulfite sequencing, using another NR probe prepared with tumor DNA after amplification with Phi29 DNA polymerase, *etc*. It is clear from Figure 1 that deleted *Not*I sites will give no signal, the same as methylated, *i.e.*, will be green whereas amplified DNA would give stronger red signal.

Hybridization of coupled *Not*I samples was carried out at 42 °C for 15 h in a Lucidea Base device (Amersham Pharmacia Biotech) according to manufacturer’s recommendations. Microarrays were scanned in a GenePix 4000A. The results were processed with GenePix Pro 6.0 software (Amersham Pharmacia Biotech). Then data were analyzed using our program NIMAN (NotI-Microarray ANalysis, see [117]).

### 4.4. Statistical Analysis

Fisher’s exact test and χ^2^ criteria were used for analysis of methylation changes in ovarian cancer groups with different histological characteristics. *p*-Values < 0.05 were considered as statistically significant. All statistical procedures were performed using NIMAN [117] and BioStat software [118]. Sensitivity was calculated as the proportion of true positives that were correctly identified by the set. Specificity was calculated as the proportion of true negatives that were correctly identified by the set [119].

### 4.5. PCR, Cloning, Bisulfite Sequencing

PCR, cloning, bisulfite sequencing were done as described earlier [120].

## 5. Conclusions

We selected novel epigenetic markers including *NKIRAS1/RPL15*, *THRB RBPS3* (*CTDSPL*), *IQSEC1*, *NBEAL2*, *ZIC4*, *LOC285205*, *CGGBP1*, *EPHB1* and *FOXP1* that allowed detection of cancer in ovarian biopsies on all stages with sensitivity equal to (72 ± 11)% and specificity (94 ± 5)%. This set allowed us to discriminate between Stages I+II and III+IV with sensitivity equal to (80 ± 13)% and specificity (88 ± 12)%. We will confirm this with a validation set of new EOC samples and add markers from other chromosomes. Among eleven genes included in our set, there are six genes for which no information about their involvement in ovarian carcinogenesis have been shown: *NKIRAS1/RPL15*, *THRB*, *IQSEC1*, *NBEAL2* and *ZIC4*. For the 5 remaining genes except *FOXP1* only slightly relevant information was published.

## Figures and Tables

**Table 1:** Methylation/deletion frequencies for 35 genes with the highest percent of changes in ovarian cancer.

No.	*Not*I-site	Gene	Locus	Event frequency, (%)	

				Met/Del	Met/Del without no info
1	NR1-XM13C	*IQSEC1*	3p25.2	33 (6/18)	38 (6/16)
2	NL1-CJ4R (C)	*NKIRAS1/RPL15*	3p24.2	33 (6/18)	35 (6/17)
3	NL4-BB6R (C)	*THRB*	3p24.2	33 (6/18)	38 (6/16)
4	NL3-CA11RS	*LRRC3B*	3p24	33 (6/18)	35 (6/17)
5	NLJ-003RD	*RBSP3*(*CTDSPL*)	3p21.3	33 (6/18)	33 (6/18)
6	NR1-KA8R (C)	*THRB*	3p24.2	28 (5/18)	56 (5/9)
7	NL1A401R (D)	*ITGA9*	3p21.3	28 (5/18)	29 (5/17)
8	NL3A006R (D)	*NBEAL2*	3p21.31	28 (5/18)	33 (5/15)
9	NL3A001R (D)	*GNAI2*	3p21.31	28 (5/18)	28 (5/18)
10	NL1-DE18R	*GATA2*	3q21.3	28 (5/18)	28 (5/18)
11	NL4-BH3R (C)	*GATA2*	3q21.3	28 (5/18)	28 (5/18)
12	NR1-PD1R	*ZIC4*	3q24	28 (5/18)	31 (5/16)
13	NL3003R (U)	*GORASP1/TTC21A*	3p22–p21.33	22 (4/18)	24 (4/17)
14	NR1-AN24RS	*ABHD5/C3orf77*	3p21	22 (4/18)	22 (4/18)
15	NR1-WE11RS	*CGGBP1*	3p12–p11.1	22 (4/18)	24 (4/17)
16	NL3-CI2R (C)	*LOC285205*	3p13.12	22 (4/18)	27 (4/15)
17	NR1-WD21R (C)	*NEK11/NUDT16*	3q22.1	22 (4/18)	44 (4/9)
18	NR5-IO11R (C)	*PAQR9*	3q23	22 (4/18)	25 (4/16)
19	NR1-AK24R	*BCL6*	3q27	22 (4/18)	24 (4/17)
20	NL6-FJ5R (C)	*LRRN1*	3p26.2	17 (3/18)	17 (3/18)
21	NR1-KJ5R (C)	*FBLN2*	3p25.1	17 (3/18)	19 (3/16)
22	NR1-PL22R (C)	*LOC285375*	3p25.1	17 (3/18)	18 (3/17)
23	NL4-BK12R (C)	*WNT7A*	3p25	17 (3/18)	18 (3/17)
24	NL1308R (D)	*MOBP*	3p22.1	17 (3/18)	19 (3/16)
25	NR1-NC7RS	*PPM1M*	3p21.2	17 (3/18)	19 (3/16)
26	NR1-NJ9R (C)	*PRICKLE2*	3p14.1	17 (3/18)	18 (3/17)
27	NL1-BA6R	*FOXP1*	3p14.1	17 (3/18)	17 (3/18)
28	NL6-F020R (C)	*DCBLD2*	3q12.1	17 (3/18)	19 (3/16)
29	NL1-GK21R (C)	*ROPN1/KALRN*	3q13.3	17 (3/18)	21 (3/14)
30	NL1290R (D)	*CHST13*	3q21.3	17 (3/18)	17 (3/18)
31	NL2A230R	*ABTB1/PODXL2*	3q21	17 (3/18)	17 (3/18)
32	NL4-DJ11RS	*TRH*	3q13.3–q21	17 (3/18)	18 (3/17)
33	NL1A079R (D)	*EPHB1*	3q21–q23	17 (3/18)	19 (3/16)
34	NL1-FK10R (C)	*PPP2R3A*	3q22.1	17 (3/18)	21 (3/14)
35	NR1-NH1R (C)	*FGF12*	3q28	17 (3/18)	18 (3/17)

**Table 2:** Methylation/Deletion frequency for eight genes in two groups of samples.

Gene	Methylation/Deletion frequency, %		*p*-Parameter *

	Stages I + II	Stages III + IV	
*LOC285205*	0 (0/7)	50 (4/8)	*0.08*
*CGGBP1*	0 (0/7)	40 (4/10)	*0.10*
*EPHB1*	0 (0/7)	33 (3/9)	*0.21*
*FOXP1*	0 (0/8)	30 (3/10)	*0.22*
*WNT7A*	0 (0/7)	30 (3/10)	*0.23*
*NKIRAS1/RPL15*	14 (1/7)	50 (5/10)	*0.30*
*GATA2*	13 (1/8)	40 (4/10)	*0.31*

Note:

* *p*-parameter calculated using Fisher’s exact test.

**Table 3:** Early detection and discrimination of ovarian cancer groups with different histological characteristics using the set of 10 selected markers.

Use	Sets of markers
Early detection	*NKIRAS1/RPL15*, *THRB*, *RBPS3* (*CTDSPL*), *IQSEC1*, *NBEAL2*, *ZIC4*, *LOC285205*, *FOXP1* Sp = (94 ± 5)% Sn = (72 ± 11)% *p* < 0.01
Discrimination of BOA and EOC	*NKIRAS1/RPL15*, *THRB*, *RBPS3* (*CTDSPL*), *IQSEC1*, *NBEAL2*, *ZIC4*, *LOC285205*, *FOXP1* Sp = (71 ± 17)% Sn = (72 ± 11)% *p* = 0.04
Discrimination of Stages I + II and Stages III + IV	*LOC285205*, *CGGBP1*, *EPHB1*, *NKIRAS1/RPL15* Sp = (88 ± 12)% Sn = (80 ± 13)% *p* < 0.01

Note: Sp, specificity; Sn, sensitivity of the set. *p*-Parameter shows significance of compared groups distinction, calculated using Fisher exact test and χ^2^ criteria.

**Table 4:** Annotations for gene markers involved in ovarian cancer and their protein products.

Gene symbol and location	Protein Function	Involvment in cancer
*LOC285205* 3q13.12	This gene encodes uncharacterized protein with moderate expression level in ovary, low level in brain, bladder, skin, breast, and testis (according to the dbEST and SAGE). Rather high expression level of this gene is observed in ovarian normal tissue (^*^).	Only EST and SAGE data is available. According to this, expression level in ovary, testis and some types of brain tumors is expected to be decreased.
*CGGBP1* 3p12–p11.1	Binds to unmethylated 5′-d(CGG)(*n*)-3′ trinucleotide repeats in the FMR1 (fragile X mental retardation gene) promoter and the ribosomal RNA gene clusters. Regulates *FMR1* gene expression. Regulates gene expression during heat shock stress response. CGGBP1 is known to be a cell cycle regulatory midbody protein required for normal cytokinetic abscission in normal human fibroblasts (^*^).	Decreased mRNA level in testis cancer and various cell lines [23,24]. Microsatellite instability in ovarian cancer cell line [25]. The role of CGGBP1 in cell cycle involves multiple mechanisms: depletion of *CGGBP1* mRNA observed in tumor cells leads to increase of the expression of cell cycle regulatory genes *CDKN1A* and *GAS1*; otherwise, a presence of CGGBP1 is required for the ability of cancer cells to progress cell cycle beyond G_0_/G_1_ [24].
*EPHB1* 3q21–q23	Encodes a member of attractive and repulsive axon-guidance molecules family (that includes SEMA5A, in addition); mediates numerous developmental processes, particularly in the nervous system. Receptor for members of the ephrin-B family. Binds to ephrin-B1, -B2 and -B3. Binding with the guidance cue ephrin-B2 at the optic chiasm midline redirect ventrotemporal (VT) retinal ganglion cells (RGCs) axons ipsilaterally. May be involved in cell-cell interactions in the nervous system (^*^)	Involvement in bone cancer pain [26,27]. Aberrant DNA methylation and epigenetic inactivation in acute lymphoblastic leukemia [28]. Underexpressed in poorly differentiated colorectal cancers [29]. Loss of expression in gastric carcinoma associated with invasion and metastasis [30]. Up-regulation in rhabdomyosarcoma [31]. Transduces signals to activate integrin-mediated migration, attachment and angiogenesis [32]. Expression level alterations in ovarian cancer [33]
*FOXP1* 3p14.1	FOXP1 belongs to the family of Forkhead box proteins, which contain a common DNA-binding domain termed the forkhead box or winged helix domain. FOXP1 is involved in the negative regulation of tissue- and cell type-specific gene transcription. FOXO1 and FOXP1 also have regulatory function in recombination activating gene 1 (RAG) expression in cancer cells [34].	*FOXP1* has been reported to be associated with development of various types of tumors. Involved in chromosomal translocation in MALT lymphoma [35,36] and in large B-cell lymphoma [37]. Deletions, both mRNA and protein down-regulation in a wide range of tumors [38]. LOH and copy number alterations in kidney cancer [39]. Highly expressed in a subset of B-cell lymphoma [40]. Down-regulated in endometrial cancer [41]. High expression of tumor-specific smaller isoforms in B-cell lymphoma and Follicular lymphomas [42,43]. *FOXP1* is located in the chromosomal region 3p14.1 reported to contain a number of TSGs [38,44]. *FOXP1* is found to be significantly down-regulated in stage III serous ovarian carcinoma [45].
*WNT7A* 3p25	A member of the WNT gene family, which consists of structurally related genes that encode secreted signaling proteins. These proteins have been implicated in oncogenesis and in several developmental processes, including regulation of cell development and patterning during embryogenesis. WNT7A binds to the Fzd9 receptor and signals through ERK-5 to activate the tumor suppressor peroxisome proliferator-activated receptor γ (PPARγ) [46]. PAPRγ inhibits transformed cells growth and metastasis and promote epithelial differentiation and have demonstrated tumor prevention efficacy [47,48].	Methylation of *WNT7A* promoter modulated with DNMT1 has been reported for non-small cell lung cancer [49]. It was shown that WNT7A regulates tumor growth and progression in ovarian cancer through the WNT/β-catenin pathway abnormally activated in ovarian cancer. Abundant WNT7A was found in the epithelium of serous ovarian carcinomas, but not detected in borderline and benign tumors, normal ovary, or endometrioid carcinomas [50]. Down-regulation in lung cancer [51,52], in uterine leiomyoma [53]. Overexpression in thyroid cancer [54], in ovarian cancer, associated with poor prognosis [55,56]. Differential expression (down-regulation), associated with poor prognosis in head and neck squamous cell carcinoma [57].
*NKIRAS1/RPL15* 3p24.2	*NKIRAS1*: Atypical Ras-like protein that acts as a potent regulator of NF-κ-B activity by preventing the degradation of NF-κ-B inhibitor beta (NFKBIB). Both GTP- and GDP-bound forms block phosphorylation of NFKBIB (^*^) *RPL15*: A ribosomal protein that is a component of the large 60S subunit. The protein belongs to the L15E family of ribosomal proteins. Transcript variants utilizing alternative polyA signals exist. Interacts with IFIT1 [58]; up-regulation of both *IFIT1* and *RPL15* may lead to proliferative inhibition of gastric cancer cells [58].	*NKIRAS1*: Chromosomal aberrations and subsequent down-regulation in kidney cancer; furthermore, high grade kidney tumors (III and IV stage) revealed lower *NKIRAS1* mRNA level than low grade ones (stage I and II) [59]. Overexpression, associated with poor prognosis in gliomas [60]. *RPL15*: Overexpression in gastric cancer [61]. Differentially expressed in cutaneous squamous cell carcinoma [62].
*GATA2* 3q21.3	This gene encodes a member of the GATA family of zinc-finger transcription factors. GATA proteins bind the DNA sequence WGATAR and, along with other cofactors, drive expression of target genes important in development of a variety of tissues [63]. For example, the encoded protein plays an essential role in regulating transcription of genes involved in the development and proliferation of hematopoietic and endocrine cell lineages, e.g., activation of beta-thyrotropin (thyroid-stimulating hormone) expression [64].	*GATA2* along with *ZIC4* was found to have methylated CpG islands in bladder cancer [65]. *GATA2* mutations are associated with hereditary myelodysplastic syndrome and extreme risk of acute myelogenous leukemia development [66,67]. Considering murine model, *GATA2* promoter methylation was found to be associated with development of breast cancer (BC); its down-regulation was seen for human BC [68]. However, *GATA2* negatively regulates *PTEN* (phosphatase and tensin homolog deleted on chromosome 10) tumor suppressor by preventing nuclear translocation of androgen receptor and by androgen-independent suppression of *PTEN* transcription in breast cancer [69].
*THRB* 3p24.2	Encodes receptor of nuclear hormone receptor for triiodothyronine. The thyroid hormone receptors (TRs) are transcription factors that mediate the pleiotropic activities of the thyroid hormone, T3. TRs regulate cell proliferation, differentiation, and apoptosis [70]. These TRs are expressed in a tissue-dependent and developmentally regulated manner. Different hormone receptors, while having certain extent of redundancy, may mediate different functions of thyroid hormone. *THRB* acts as a tumor suppressor and disturbances of the *THRB* gene are frequent findings in cancer [71].	In mouse models, a truncated THRB gene leads to thyroid cancer (TC); it can be down-regulated at least with seven miRNAs overexpressed in papillary TC [70]. *THRB* aberrant methylation can be found in tissue and plasma of BC patients [72]. *THRB* revealed a low frequency of methylation in prostate cancer samples [73], but high frequency of LOH in prostate [74,75], esophageal cancer [76], endocrine tumors of the cervix [77], head and neck cancer [78]; also small LOH frequencies were shown for NSCLC [79]. Mutation of this gene in mice predisposes to the development of mammary tumors [80]. Reduced *THRB* expression was shown for clear cell renal cell cancer samples which can be resulted from regulatory effects of *THRB* 5′ and 3′ UTRs on THRB protein translation [71].
*RBPS3* (*CTDSPL*) 3p21.3	*RBSP3/CTDSPL* belongs to a gene family of small CTD phosphatases that preferentially catalyzes serine-5 dephosphorylation in the specific sequence of the RNA polymerase II (Pol II) large subunit and in other proteins. This leads to inactivation of Pol II and negative regulation of transcriptional activity. RBSP3 is thought also to activate RB1 (retinoblastoma 1) tumor suppressor precursor, that leads to cell cycle arrest at G_1_/S phases boundary. *RBSP3* is TSG whose product is likely to be an important component of the Rb cycle regulation pathway. *RBSP3* transcribes two isoforms with antitumor activity, which is more pronounced for the product of isoform B [81].	*RBSP3* showed a low expression level because of deletions and methylation in various epithelial tumors [82–86]; these aberrations were also found in early dysplastic lesions of head and neck [87], premalignant cervical lesions [88]. *RBSP3* gene revealed high mutability rate in various primary tumors and cell lines [89]; tumor suppressor activity revealed for lung and renal cancer cell lines ACC-LC5 and KRC/Y, *in vitro* and *in vivo* [81]; transient protein expression resulting in a significant decrease of phosphorylated RB1 level, which may lead to cell cycle arrest between G_1_ and S phases [81]. Acute myeloid leukemia reveals specific overexpression of mir-100 targeting *RBSP3*, which promotes cell proliferation and blocks granulocyte/monocyte differentiation [90].
*IQSEC1* 3p25.2	The representative of guanine-exchange proteins binding to ADP-rybosylation factors. This protein preferentially works as a guanine nucleotide exchange protein for ARF6 (ADP-ribosylation factor), a member of a family of small GTPases, mediating internalization of beta-1 integrin [91]. Regulates phagocytosis of monocytic phagocytes [92].	The EGFR-IQSEC1-ARF6-AMAP1 signaling pathway is essential for breast cancer (BC) invasion and metastasis. Overexpressed IQSEC1 is responsible for activation of ARF6 which leads to BC invasion and metastasis. IQSEC1, in turn, is activated by ligand-dependent epidermal growth factor receptor (EGFR) [93,94].
*NBEAL2* 3p21.31	Encodes a BEACH/ARM/WD40 domain protein. Mutations in this gene are leading to gray platelet syndrome (a rare congenital bleeding disorder caused by a reduction or absence of alpha-granules in blood platelets) [95]. NBEAL2 protein is predicted to interact with WDFY3 (WD repeat and FYVE domain containing 3), which itself interacts with CHS1, and with DLL1 and JAG1 [96], known to have roles in hematopoiesis.	The gene is located in close proximity to the LUCA and AP20 regions subject to frequent aberrations in various tumors, but there are no literature data concerning such *NBEAL2* alterations. GeneNote, EST and SAGE analysis reveal omnipresent expression character of *NBEAL* and allow to expect its probable mRNA level decreases in thymus, brain (various type of tumors), liver, pancreas, prostate cancer and leukemia (^*^).
*ZIC4* 3q24	Encodes a member of the ZIC family of C2H2-type zinc finger proteins. Members of this family plays important roles during development, and have been associated with X-linked visceral heterotaxy and holoprosencephaly type 5; heterozygous deletion of *ZIC4* are associated with Dandy-Walker cerebellum malformation syndrome [97].	*ZIC4* along with *GATA2* was found to have methylated CpG islands in bladder cancer; it was associated with high extent of progression and invasive character of bladder tumors [65]. Zic4 along with Zic1–5, other members of this family, was shown to suppress β-catenin-mediated transcriptional activation within the Wnt/β-catenin signaling pathway (in *Xenopus laevis*). ZIC1, ZIC2, and ZIC5 were found to be novel molecular markers for meningiomas whereas ZIC4 expression is highly selective for medulloblastomas [98]. Using NotI-microarrays, *ZIC4* aberrant methylation/deletions were found for various types of tumors [84]. Consistent up-regulation of the neural transcription factors *ZIC1* and *ZIC4* was shown for desmoid tumors and other fibroproliferative disorders [99]. *ZIC4* aberrations are associated with paraneoplastic neurologic disorders and small-cell lung cancer [100,101].

Note: asterisks (^*^) indicate these data were obtained from GeneCards web portal [102].
